# COMMunity of Practice And Safety Support for Navigating Pain (COMPASS-NP): study protocol for a randomized controlled trial with home care workers

**DOI:** 10.1186/s13063-023-07149-8

**Published:** 2023-04-10

**Authors:** Ryan Olson, Jennifer A. Hess, Dennis Turk, Miguel Marino, Leah Greenspan, Lindsey Alley, Courtney Donovan, Sean P.M. Rice

**Affiliations:** 1 Oregon Institute of Occupational Health Sciences, Oregon Health & Science University, 3222 SW Research Drive, Portland, OR 97239-3098 USA; 2 Oregon Health & Science University-Portland State University School of Public Health, VPT, 3181 SW Sam Jackson Park Road, Portland, OR 97239 USA; 3 Department of Psychology, Portland State University, P.O. Box 751, Portland, OR 97207-0751 USA; 4 Labor Education & Research Center, University of Oregon, 1675 Agate Street, Eugene, OR 97403-1289 USA; 5 Department of Anesthesiology & Pain Medicine, University of Washington, BB 1425 HSC, Box 356540, 1949 NE Pacific Street, Seattle, WA 98195-6540 USA; 6 Family Medicine, School of Medicine, Oregon Health & Science University, FM, 3181 SW Sam Jackson Park, Portland, OR 97239 USA

**Keywords:** Home care workers, Total Worker Health, Pain management, Ergonomics, Cognitive-behavioral therapy, Injury prevention, Self-management

## Abstract

**Background:**

Chronic pain is a prevalent and costly problem that often has occupational origins. Home care workers (HCWs) are at high risk for work-related injuries, pain, and disability. Current treatments for chronic pain emphasize medications, which are an inadequate stand-alone treatment and can produce significant adverse effects.

**Methods:**

In this translational study, we will adapt an established work-based injury prevention and health promotion program (COMmunity of Practice And Safety Support: COMPASS) to address the needs of HCWs experiencing chronic pain. COMPASS employs peer-led, scripted group meetings that include educational content, activities, goal setting, and structured social support. The translated intervention, named COMPASS for Navigating Pain (COMPASS-NP), will be delivered in an online group format. Safety protections will be strengthened through an ergonomic self-assessment and vouchers for purchasing ergonomic tools. Educational content will integrate a self-management approach to chronic pain using proven cognitive-behavioral therapy (CBT) principles. We will use a mixed-methods hybrid type 2 evaluation approach to assess effectiveness and implementation. A cluster-randomized waitlist control design will involve 14 groups of 10 HCWs (*n* = 140) recruited from Washington, Oregon, and Idaho. Half of the groups will be randomly selected to complete the intervention during the first 10 weeks, while the waitlist groups serve as controls. During weeks 10–20, the waitlist groups will complete the intervention while the original intervention groups complete a follow-up period without further intervention. Our primary hypothesis is that COMPASS-NP will reduce pain interference with work and life. Secondary outcomes include injury and pain prevention behaviors, pain severity, changes in medication use, risk for opioid misuse, well-being, physical activity, and sleep. Qualitative data, including phone interviews with group facilitators and organizational partners, will evaluate the implementation and guide dissemination.

**Discussion:**

The results will advance the use and knowledge of secondary prevention interventions such as ergonomic tools and cognitive behavior therapy, to reduce injury, pain, and disability and to encourage appropriate uses of analgesic medications among HCWs.

**Trial registration:**

ClinicalTrials.gov NCT05492903. Registered on 08 August 2022

**Supplementary Information:**

The online version contains supplementary material available at 10.1186/s13063-023-07149-8.

## Background

Chronic pain is a prevalent problem impacting many people’s lives. According to the International Classification of Diseases of the World Health Organization, chronic pain is defined as persistent or recurrent pain lasting longer than 3 months [1]. Chronic pain, defined as pain that is experienced every day for the preceding 3 months or more, affects 11.2% of US adults, and 41% of those with daily pain report the pain is severe [2]. In addition to the suffering and losses experienced by those affected, there are impacts on family and friends, as well as societal costs in compensation payments and lost productivity [3]. Common treatments for chronic pain emphasize analgesic medications, regional anesthesia, and surgeries. These treatments have significant risks and side effects, and their effectiveness as stand-alone tactics is limited [4, 5]. However, when coupled with pain self-management education and cognitive-behavioral therapy (CBT), treatment effectiveness increases [6]. Although such tertiary prevention approaches can provide substantial benefits, they can be costly and require a substantial time commitment from patients (e.g., full-day programs extended for up to 6 weeks), and there is limited availability. There are important opportunities for earlier interventions. For people who work full time, the workplace represents at least 50% of their exposures to physical injury and pain-inducing hazards [7]. The current project focuses on a work-based secondary prevention approach to address and interrupt the progression of pain and associated problems within an at-risk working population.

Home care workers (HCWs) are a growing and vulnerable working population that bears a great burden of pain and associated problems. There are an estimated 3.6 million home care workers (HCWs) and personal care aides in the USA, and the occupation is expected to grow 25% by 2030 [7]. HCWs are predominantly low-income, older (mean age mid-40 s), female, increasingly immigrants, people of color, and ethnic minorities [8]. Common home care tasks pose musculoskeletal injury hazards when performed alone or without ergonomic equipment or tools and include bathing, transferring, and transporting clients and performing housekeeping tasks (e.g., making beds, scrubbing walls/floors) [9]. In 2020, the lost time injury rate for home health and personal care aides was 166.9 per 10,000 full-time workers, compared to 120.7 for all occupations. Lumbosacral pain is prevalent among HCWs, and those exposed to client moving/transferring tasks also experience chronic pain in their upper extremities [9]. HCWs reported in 2020 a rate of general soreness and pain that was nearly double that of all occupations combined (27.0 vs. 15.4 per 10,000 full-time workers) [10]. As a financial necessity, the majority of HCWs with injuries or pain continue to work [11], placing them at risk for exacerbations of pain, re-injury, worsening chronic pain, related disability, and opioid misuse.

Surveillance research with HCWs in Washington state investigated the prevalence of pain, workers’ pain management strategies, and opioid misuse risk. In that study, 54.2% of respondents met the study-specific criteria for elevated pain (i.e., self-reported experiences of pain worse than normal in the last week or perceived need for daily pain medication) [12]. Over-the-counter medications were the predominant pain management strategy reported by 67.3% of HCWs in the sample, with 4.8% reporting current prescription opioid use. Elevated pain, as well as several biopsychosocial factors (e.g., injuries, financial strain, depressive symptoms), was significantly associated with opioid misuse risk [12]. Opioid use, and potential misuse, in this middle-aged population is a priority topic. Between 1999 and 2015, this demographic experienced a staggering 471% increase in prescription opioid-related overdoses.

There is a clear need for practical resources and interventions for HCWs experiencing chronic pain that address their uniquely demanding work exposures and barriers. HCWs are an isolated and dispersed workforce and have access to fewer occupational safety and health protections and supports than more typical workers, such as safety committees, hazards assessments, ergonomic tools, physical assistance from co-workers, and supervision. Moreover, HCWs’ workplaces are their clients’ private residences. Clients are rarely aware of safety standards or the importance of ergonomic equipment (e.g., mechanical lifts) and tools (e.g., slide boards) for protecting workers [13]. Qualitative research indicates that HCWs face many barriers to acquiring ergonomics tools, forcing them to purchase their own tools, improvise with potentially dangerous homemade tools, or do without [14]. Assessing task demands, tool needs, and reducing barriers to accessing ergonomic assistive devices are important work-based areas for primary and secondary injury and pain prevention interventions [12].

When primary prevention fails and injury occurs, work-based injury rehabilitation and return-to-work programs are important for reducing disability and improving functioning [15]. However, dominant approaches to rehabilitation provide support only after problems have become severe and emphasize physical functioning and outcomes like absence and productivity [16–20]. Both return-to-work programs and medication-only treatments tend to neglect important causal drivers of impaired functioning such as pain severity, emotional distress, and pain interference with multiple work and life domains and also underemphasize the role of workers’ cognitive and behavioral strategies for managing their symptoms.

Alternative and complementary approaches promoting the management of chronic pain through non-pharmacological strategies have been established as a public health priority by the United States Department of Health and Human Services and the National Academies of Sciences, Engineering, and Medicine [21, 22]. The Centers for Disease Control and Prevention and Department of Health and Human Services [21] have called to broaden the scope of chronic pain treatment and coverage to include methods, such as cognitive-behavioral therapy (CBT) for pain management, in part to reduce pain progression, disability, and prescription opioid use and misuse [23]. The workplace presents an important and potentially powerful locus for interventions of this type; however, the few work-based pain management interventions to integrate CBT have lacked strong safety and ergonomic protections that are fundamental for injury and pain prevention [24–26]. None has extended CBT to HCWs with chronic pain.

### Rationale

To address the problems of work-related injury, pain onset and exacerbation, and potential medication misuse among HCWs, the current project will translate an established intervention for injury prevention developed for HCWs to address the special needs of those who are experiencing chronic pain. The original COMmunity of Practice And Safety Support (COMPASS) intervention, created and evaluated within the Oregon Healthy Workforce Center (OHWC), is a group program that employs peer-led, scripted meetings. A professional trainer or facilitator conducts the initial meeting to demonstrate the process, and then workers take turns serving as peer leaders. Meetings include educational content (i.e., diet, exercise, use of low-tech ergonomic tools, and client communication), activities, goal setting, and structured social support [27–29]. Clusters of HCWs (16 clusters, *n*= 149 workers) were randomized to intervention and control conditions. The intervention involved 12 monthly peer-led and scripted meetings. Evaluation data were collected at baseline, 6 months (mid-intervention), and 12 months (post-intervention). Relative to control, the intervention produced improvements to injury protective safety practices and included the use of ergonomic tools or techniques for physical work, safety communication with clients, and hazard correction in homes. Injury-related lost work days also significantly reduced [28].

The COMPASS study did not directly address chronic pain management, and consequently, reductions in pain severity were small and non-significant [27]. Our qualitative analysis revealed there were persistent barriers for HCWs to access ergonomic tools that are typically not covered by insurance (e.g., slide boards, long-handled cleaning tools). We also found that obtaining tools is exhausting and stressful for low-wage HCWs, who are often forced to buy their own, improvise with untested homemade tools, or do without [14]. To explore the potential differential effects for HCWs with pain in the original COMPASS trial, secondary analyses were conducted with intervention group participants focused on safety and mental health outcomes. In a generalized linear mixed model, HCWs with pain limitations [30] at baseline showed a trend (*F*[1,60] = 2.11, *p* = 0.15) of making larger 6-month increases in the mean safety actions (Cohen’s *d* = 0.75) compared to those without limitations (Cohen’s *d* = 0.44; difference-in-difference Cohen’s *d* = 0.31). However, workers with pain limitations experienced a trend (*F*[1,57] = 0.73, *p* = 0.40) of worsening mental health [31] (Cohen’s *d* =  − 0.08) compared to improvements (Cohen’s *d* = 0.11) experienced by those without (difference-in-difference Cohen’s *d*= 0.19). One interpretation is that workers with pain tried harder to achieve pain relief, but without pain education and CBT pain management strategies, they experienced less intervention benefit for mental health. The lack of pain-specific content, barriers to accessing tools, and differential outcomes for HCWs with pain highlight the need for a translated version of COMPASS. Therefore, COMPASS for Navigating Pain or COMPASS-NP will be implemented with virtual web-based groups to maximize availability, efficiency, retention, and dissemination potential. The intervention translation plan includes strengthening ergonomic protections, adapting established educational content for workers experiencing chronic pain, and integrating new content focused on pain self-management and CBT strategies [6, 32, 33].

Theoretical and conceptual foundations of the base [34] and planned translated intervention include community of practice theory [35], the social cognitive theory of self-regulation [36], reinforcement or operant learning theory [37], and *Total Worker Health*® approaches to workplace interventions [38]. COMPASS-NP will expand these foundations to include CBT strategies for pain self-management. The cognitive-behavioral perspective and techniques outlined in CBT for pain management [39] are aligned with the biopsychosocial model of pain [40]. CBT for pain management is specifically designed to build perceived control and self-efficacy for managing pain [41]. Components of CBT have been effective in prior workplace interventions [25, 42, 43], with lower educated individuals [44, 45]. Research supports the efficacy of CBT for improving physical and emotional functioning and reducing pain-related disability [42, 46]. CBT is often combined with other strategies to bolster effectiveness. For example, when combined with exercise [47, 48] and sleep hygiene interventions, CBT produced significantly better pain outcomes relative to control or single-strategy conditions. These perspectives and strategies focus on altering individuals’ appraisal of their pain, interpretation of causes and responses, and advancing skills and self-efficacy for managing pain. These emphases are designed to influence individuals’ expectations, acceptance of treatment and their role in pain self-management, adherence to and responses to treatment (e.g., improved physical and emotional functioning, reduced pain interference with life, reduced dependence on pharmacologics) [49]. Specific skills are taught in CBT that center on pain self-management strategies, such as goal setting, problem solving, self-monitoring of pain and coping, physical activity pacing, and prevention through protective and healthy lifestyle practices.

The current study is a translational research project within the OHWC – a NIOSH Center of Excellence for *Total Worker Health*(TWH). The Center’s current theme is “intervention effectiveness, translation, and outreach to advance safe and healthy work design.” TWH is defined by NIOSH as “policies, programs, and practices that integrate protection from work-related safety and health hazards with promotion of injury and illness-prevention efforts to advance worker well-being” [38].

Our primary outcome, pain interference, is a widely used target in pain intervention research. It is inclusive of pain-related physical limitations but also addresses pain interference with additional work-life domains (e.g., relationships, sleep, enjoyment of life). Thus, our primary hypothesis is that COMPASS-NP will have more reduction in pain interference with work and life in the intervention group relative to the control group. Our secondary outcomes will evaluate injury and pain prevention behaviors (e.g., ergonomic tool use), occurrence of injuries, pain severity, changes in medication use, risk for opioid misuse, worker well-being, and objective measures of physical activity and sleep (i.e., actigraphic). Implementation measures will explore the process differences across partners’ systems and assess the intervention fidelity and acceptability. Our secondary hypotheses assert that these outcomes will change in favorable directions relative to the control group. These measures will include environmental and system characteristics, HCW attendance and participation in group meetings, HCW and facilitator characteristics and qualitative feedback, and HCW ratings of the acceptability of the goals, processes, and outcomes of the intervention.

## Methods/design

The goals of our translational intervention research are to provide HCWs with an effective work-based program to prevent injuries/re-injury, build pain self-management skills, and reduce pain interference with work and life. To maximize intervention reach and dissemination potential, COMPASS-NP will be implemented and evaluated in an efficient Internet-based format. Original COMPASS materials will be enhanced with the addition of an online ergonomic self-assessment and a voucher for purchasing tools. We will also adapt existing educational modules to address the needs and limitations of HCWs with elevated pain and create new specific educational modules focused on CBT strategies for pain self-management based on *The Pain Survival Guide *[50]*.* Our specific aims are to (1) pilot COMPASS-NP in Oregon, (2) determine the effects of COMPASS-NP across three states (i.e., Oregon, Washington, and Idaho), (3) describe the translation and implementation of COMPASS-NP across partners and systems, and (4) disseminate COMPASS-NP knowledge, tools, and toolkits.

This protocol has been written according to the recommendations of the SPIRIT 2013 statement [51]. SPIRIT provides guidelines that define the standard elements of a protocol, including recommendations for intervention trials. A completed checklist can be found in Additional file 1: SPIRIT Guidelines.

### Participants, recruitment, randomization, and statistical power analysis

HCWs (*n* = 140) with the presence of symptoms of chronic pain will be recruited across Oregon, Washington, and Idaho to participate in this study with the help of our partner organizations: the Oregon Home Care Commission, the Service Employees International Union (SEIU) 775 Benefits Group in Washington, and the St. Luke’s Health System in Idaho. We will perform 1:1 randomization of block size 2 within each state. The randomized groups will be computer generated by the study statistician and kept in sealed envelopes.

**Fig. 1:**
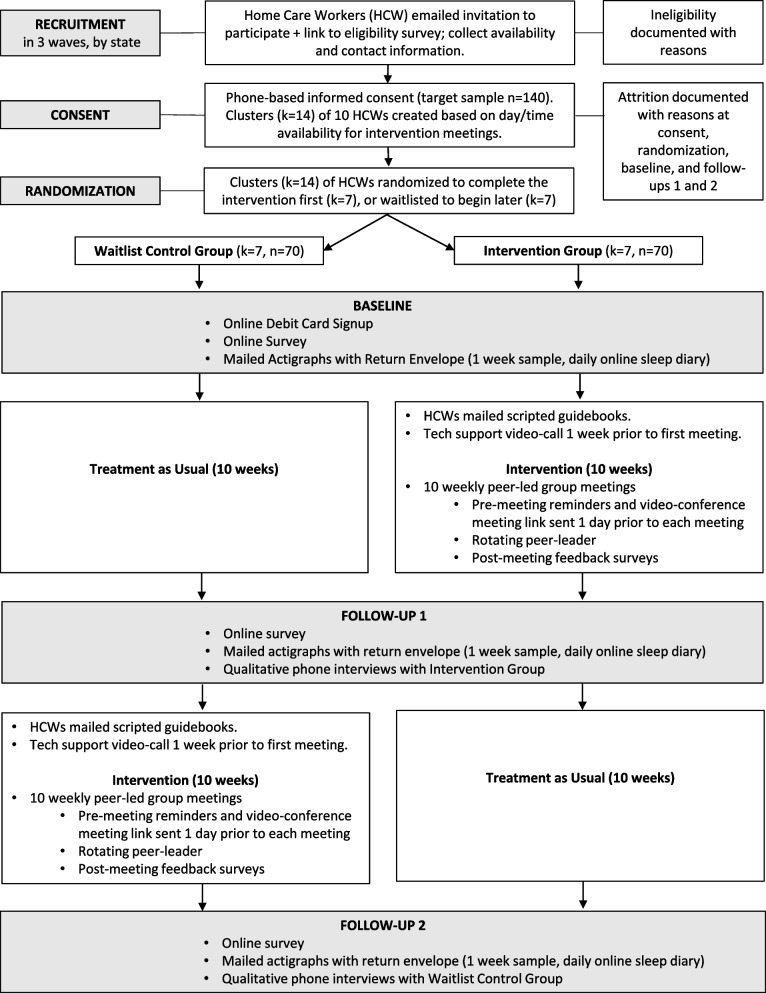
Participant timeline

The effectiveness and implementation of COMPASS-NP will be evaluated using a mixed-methods hybrid type II evaluation approach [52]. In this cluster randomized waitlist control design, all HCWs will participate in the trial for 20 weeks, with measures collected by online surveys at baseline, 10 weeks (follow-up 1), and 20 weeks (follow-up 2) (Fig. 1). Fourteen groups of 10 workers each will be created based on residence in the same state and schedule availability for attending group meetings. Half of the recruited groups in each state will be randomly selected to complete the intervention during the first 10 weeks, while the remaining groups serve as wait-list controls (i.e., delayed start) during the same period. After 10 weeks, the wait-list groups will initiate the intervention and finish at week 20. Original intervention participants will experience usual practice during weeks 10 to 20. A randomized waitlist study design was chosen because it creates an intervention vs. control group comparison but also permits every participant to eventually receive the intervention. Given our focus on a vulnerable population in high need of interventions, we deemed this to be the best and most ethical approach for our project.

The sample size target of 140 (70 in the intervention group, 70 in the waitlist control group) was based on an a priori power analysis to achieve 80% power to detect a moderate effect size (*d*= 0.50) between the groups at 10 weeks on our primary outcomes. Our sample size requirement assumes a conservative 20% attrition at each time point (original COMPASS study saw 15% attrition at 12 months) [27]. We also assumed an AR(1) correlation structure with rho = 0.7 for repeated observations within each worker. The power calculation also takes into account a conservative intracluster correlation coefficient within each virtual group of 0.10 (in the original COMPASS trial, ICC range was 0.001–0.086) [27]. The estimated effect size of *d* = 0.50 is the average effect size for safety changes at 6 months in the original COMPASS trial, which represents a reasonable estimated effect for COMPASS-NP for pain-related limitations outcomes.

### Inclusion/exclusion criteria

The inclusion criteria will consist of adult HCWs reporting chronic pain (pain lasting 3 + months and > 4 average intensity), the current presence of pain interference with work (response of “agree” or “strongly agree” on single-item), currently working ≥ 4 h per week, and access to the Internet with a video-capable device (e.g., smartphone, tablet, or computer). The exclusion criteria will include prior exposure to the original COMPASS program, childbirth in the prior 6 months or current pregnancy, major surgery in the prior 6 months or scheduled major surgery during the study period, and any significant psychiatric problems resulting in hospitalization during the prior 6 months.

Study partner organizations will send HCW recruitment emails and also advertise the opportunity through websites, newsletters, and other established communication channels (e.g., social media). Interested HCWs will complete an online eligibility screener survey along with their schedule availability and technology comfort level. Qualifying HCWs will be contacted by the study staff by phone to discuss the study and complete an informed consent process. Once 2 groups of 10 HCWs in the same state are created, those groups will be randomized. The group receiving the intervention first will be mailed program materials and then contacted by their facilitator to schedule a pre-program call for coaching on web-based technology for group meetings.

COMPASS-NP group facilitators will be recruited from existing professional trainers or staff from partner organizations and complete training prior to leading groups (see the “ Intervention condition” section).

### Participant compensation

Facilitators and workers will be paid an hourly wage for facilitating/attending COMPASS-NP meetings (approximately $15/h for workers, $35/h for facilitators), which is consistent with our partner organization’s training practices and promotes retention. HCWs who complete the online ergonomic tool self-assessment will be provided the tools they select directly, or through a voucher, up to a value of approximately $100. HCWs will receive escalating incentives for completing surveys ($20 at baseline, $40 at 10 weeks, $60 at 20 weeks).

### Intervention condition

COMPASS-NP facilitators will complete an adapted version of an established online COMPASS Facilitator Orientation. This facilitator training describes the purpose of each meeting step followed by a video of a COMPASS facilitator and group role-playing that step. A facilitator handbook will be created to guide pre-program technical coaching of HCWs, as well as running COMPASS-NP meetings using Microsoft Teams or comparable technology. The study staff will further support facilitator onboarding by observing their initial group meetings and completing debriefing and coaching conversations afterward.

COMPASS-NP will involve 10, weekly virtual meetings (1.5 h each). Virtual meetings will be held with the Microsoft Teams video conference software or a comparable platform. HCWs will be mailed scripted guidebooks, a pain journal to track their pain and pain management behaviors, and instructions for joining virtual meetings. Facilitators will provide one-on-one technical coaching with the video conference platform with each participant prior to the first meeting, and then lead groups during that first meeting to model the leader role in the process. In subsequent meetings, facilitators will step back to monitor and support workers as they take turns serving as peer leaders. The scripted guidebook approach requires minimal training for peer leaders and group members to effectively participate. The primary mode for workers to learn the peer leader role is the scripted content for meeting one (see overview of topics below) and watching the facilitator model that role.

**Fig. 2:**
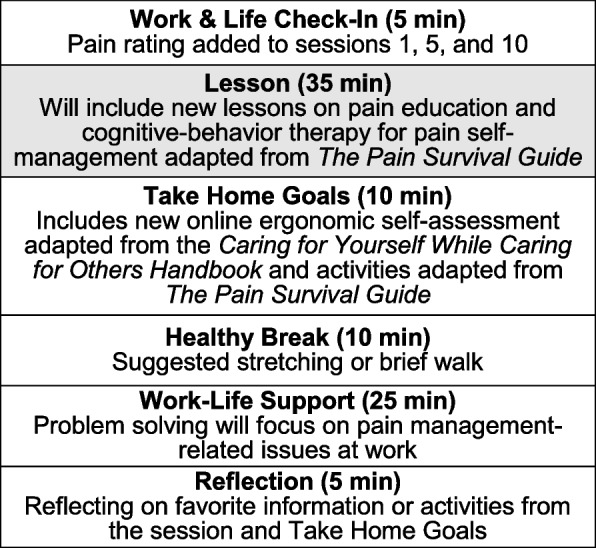
COMPASS for navigating pain meeting steps

The intervention materials for COMPASS-NP will include scripted guidebooks for 10 weekly meetings, as well as the complementary content of an online ergonomic assessment tool, online facilitator orientation training and resources, and a central program website that participants can access for resource materials. The core process and meeting steps from the original COMPASS program will be retained with adjustments such as shortened duration (90 min) and tailoring meetings for workers with chronic pain (Fig. 2).

**Fig. 3:**
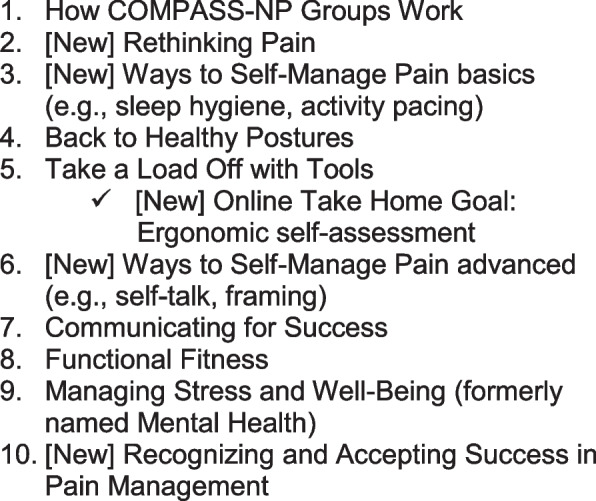
COMPASS for navigating pain meeting topics

These steps include a WorkLife check-in, an educational lesson, group and individual goal setting, a healthy break, a structured WorkLife Support process, and a meeting reflection/summary. Six existing scripted meetings addressing how COMPASS groups work (meeting 1), ergonomics (meetings 4 and 5), communication skills (meeting 7), functional fitness (meeting 8), and stress management/well-being (meeting nine) will be adapted for HCWs with chronic pain. Four new scripted lessons (Fig. 3) consisting of meetings 2, 3, 6, and 10 will address pain education and CBT for pain self-management, based on evidence-based content adapted from *The Pain Survival Guide *[50]*.*

The online ergonomic assessment tool will be adapted from the NIOSH handbook for HCWs titled *Caring for Yourself While Caring for Others* [53]. This tool will be used as a module homework assignment with follow-up and discussion in the subsequent meeting. Workers will use the tool to select their frequent work tasks from a checklist (e.g., housecleaning, client bathing, client mobility) and then be guided through educational screens based on task selections. These screens will identify the potential hazards associated with each task and methods to control them including low-tech ergonomic tools. HCWs will also receive a report, plus a list of relevant ergonomic tools to select from, and receive (up to $100 in value) based on their common work tasks (e.g., transfer clients from chair to wheelchair slide board and transfer belt).

### Intervention process and data collection

#### General process

Participants will complete online surveys that contain primary and secondary study outcomes at each study time point via REDCap [54, 55]. The post-intervention survey (10 weeks for the initial intervention group, 20 weeks for the waitlist control group) will also include ratings of the overall acceptability of intervention materials, goals, procedures, and target outcomes [56], as well as qualitative questions about the most and least helpful aspects and opportunities for improvement. Facilitators will track attendance and participation levels for each meeting, and this will be affirmed by study staff who 20% of group meetings. The research staff will also conduct interviews with partner organizations, facilitators, and a sample of HCWs to further assess the factors related to the implementation and future dissemination of the program (see the “Qualitative data” section). Participants will be emailed or texted reminders for completing surveys and attending intervention meetings. To further monitor study and intervention participation, researchers will monitor and update met or missed deadlines for study activities and then provide appropriate reminders or support.

Two of our partner organizations, the SEIU 775 Benefits Group and the Oregon Home Care Commission (OHCC), already offer well-attended live group online trainings. Attendance and a basic affirmation of participation level (e.g., did each participant take a turn reading the script) will be tracked by and collected from facilitators. We will also monitor web analytics to measure engagement with online COMPASS-NP materials (e.g., online ergonomic assessment, facilitator training videos, downloadable resources) throughout the intervention. Brief reaction measures will be collected for each meeting via REDCap using an adaptation of the OHCC training evaluation, which includes reaction ratings (affective and utility) for the lesson content, facilitator/trainer performance, and intentions to make changes to benefit the worker and their clients. Global ratings of the social validity/acceptability [57, 58] of goals, processes, and outcomes of the intervention will be collected via REDCap surveys after each group completes the intervention.

#### Quantitative data

Demographic and background survey items will include age, gender, family composition, work tenure, current work hours/clients, additional jobs (paid and unpaid/voluntary), health/injury history, current health conditions and medications, primary language, single- versus multi-income household, total income, and financial strain [59]. We will also measure several psychosocial factors known to be associated with pain and pain management, including stress [60], pain management self-efficacy [61], depressive symptoms [62], anxiety, and sleep quality [63]. We will describe the sample in total and across the participating states.

Pain interference is our primary outcome that will be measured in two domains, work and life. Pain interference is a recommended outcome in pain intervention research [64], and measuring interference with both work and life domains is consistent with integrated TWH approaches to occupational health interventions. Pain interference with work will be measured with the Work Limitations Questionnaire [65], which addresses limitations in four domains of work demands (time, physical, mental-interpersonal, and outputs). Pain interference with life will be measured with the interference subscale from the Brief Pain Inventory (BPI) [66] that addresses pain interference with general activity, walking, work, relations with other people, sleep, and enjoyment of life. The BPI is a validated 32-item measure (*α* = 0.80–0.96) [67] designed to capture a variety of factors, including pain interference, as well as the location, experience, progression, and treatment of pain [68]. It is widely used in pain outcome research [69].

Secondary outcome measures will include pain/injury prevention behaviors, injuries, pain severity, well-being, physical activity, sleep duration and efficiency, change in pain medication use, and risk of opioid misuse. Pain/injury prevention behaviors will be measured using items created in the original COMPASS research [27] that assess ergonomic tool use during housekeeping and client moving tasks and hazard correction in client homes. Injuries (first aid only and lost work time) will be reported for the past 10 weeks. Pain severity (average severity during the past week, 0 = no pain, 10 = worst pain possible) for each body region will be measured with the BPI pain severity subscale [66]. Well-being will be measured with the PROMIS Global-10 items to assess physical, mental, and social health, as well as emotional distress and overall quality of life [70]. Sleep and physical activity outcomes will be gathered using 1-week accelerometer samples at each time point (see methods below). Change in pain medication use (prescription and over-the-counter) will be assessed using a subscale from the BPI that assesses pain medication type, frequency of use (within 24 h), dependence, and concerns. The risk of opioid misuse will be measured with the Screen And Opioid Assessment For Patients With Pain—Revised (SOAPP-R) [71] for workers not currently using opioids and with the Current Opioid Misuse Measure (COMM) [72] for workers currently using opioid medications (each validated for predicting misuse).

Actigraphs (GT3x + BT, Actigraph, Pensacola, FL) will be used to obtain objective measures of physical activity and sleep. Actigraphs will be mailed to participants at each time point to collect 1-week samples of daily physical activity (waist-worn) and sleep duration/efficiency (wrist-worn). Participants will be provided with pre-paid postage envelopes to return devices to researchers. Data processing for actigraphic samples will be completed with a study-specific protocol modeled on methods used in our previously published studies [60, 73].

#### Qualitative data

Throughout the study, correspondence between the research group and partners, and between partners and participants (e.g., study advertisement communications), will be collected/documented for consideration in qualitative data analyses. During planning and coordination meetings, materials and information will be requested from organizational partners regarding their training program systems and resources (training courses offered, healthcare benefits, software platforms used for e-learning or video conferences, certification or continuing education programs and requirements, HCWs’ access to ergonomic tools). We will also request and record information from partners regarding workforce demographics, partner-specific occupational safety and health-related challenges, and plans for future resources and training that may align with COMPASS-NP adoption.

The study staff will conduct post-intervention phone interviews with group facilitators (3 to 7 expected) and organizational partner leaders (minimum 3) to gather direct feedback on the feasibility and quality of the training, perceived program sustainability and accessibility, barriers/facilitators to program adoption and participant engagement (e.g., replicating or replacing the tool provision program), challenges encountered and any innovations or strategies used to meet challenges, and additional experiences. In accordance with the occupational safety and health intervention translation model introduced by Schulte and colleagues [74], data collection will also focus on potential adopters/implementers (e.g., facilitators’ and leaders’), motivation/behavior, attitudes, organizational structures that encouraged or discouraged change, and adequacy/usability of systems within our partnering organizations. Facilitators will be invited to participate in one or more phone interviews following each wave of implementation and with organizational leaders after implementation in their state is complete. Interviews with a subsample of HCWs will also be conducted to complement qualitative data gathered from adopters and implementers. These interviews will focus on workers’ perspectives on how best to identify interested workers, advertise or market the program, and maximize ease of participation. Interview participants will be compensated $40 for each interview as permitted (government leaders may not be able to accept payment).

Interview guides and protocols will be structured based on the narrative approach to qualitative data collection [75]. The narrative interview is typically a collaborative process between interviewer and participant where both work together to generate a story—the narrator tells their story while the interviewer fosters narration through keen observation and deep listening [76]. The interviewer must be emotionally attentive and use prompts to facilitate storytelling more than answers.

### Data analysis, storage and fidelity

#### Quantitative data analysis

We will summarize participant demographics using descriptive statistics and data visualizations (e.g., histograms, scatter plots) to evaluate the differences between study arms. Effectiveness analyses will employ an intent-to-treat approach where participants are analyzed and included according to their randomized condition [77], regardless of their degree of intervention participation. This approach also utilizes all available data from participants across all time points. To test our hypothesis that the intervention group will experience reduced pain interference with work and life, we will evaluate the within- and between-group differences in the magnitude of change in the two primary outcome domains, using a difference-in-differences (DID) approach that suits the between-group and within-group design of this study. The DID approach is a general approach that can estimate relative changes in outcomes within-group and between study groups through a group-by-time interaction group. Specifically, we will implement our DID approach using generalized linear mixed models (GLMM) [78], which offer flexible regression modeling to accommodate for different sources of correlations (e.g. serial and intra-group), categorical and continuous covariates, and fixed and time-dependent covariates. These GLMMs offer a wide range of parametric distributions to model the dependent variables, including logistic regression (binary data), beta regression (percent data), Poisson regression (count data), and Gaussian regression (normally distributed data). For example, to assess the changes in pain interference, a Gaussian linear mixed model can analyze pain interference at baseline, week 10, and week 20 as a function of whether a HCW belongs to the intervention condition, time, the interaction of condition and time, and other potential confounders (e.g., psychosocial factors from our background survey). We will operationalize comparisons by computing interaction contrasts on pre/post-intervention differences within and across groups. Intervention group membership will be the between-subjects factor and pre/post-intervention assessment scores on primary outcomes will be the within-subjects factors. The interaction contrast approach will allow us to efficiently compare the 2 study arms at the end of 10 and 20 weeks on each pain interference measure with a focused, one degree of freedom hypothesis test. Additionally, to take advantage of the full longitudinal data set (baseline, 10- and 20-week time points), we will implement an analysis of response profiles to characterize the patterns of change in the mean response over time in the two arms by treating time as a categorical factor. Our approach will include random effects for virtual group clusters to account for the correlation of outcomes within virtual groups. We will also include random effects for HCWs to account for the correlation of observations within a worker over time. Depending on the distribution of the outcomes of interest, standard errors may be difficult to estimate using standard regression variance output. Thus, if we cannot accurately represent standard errors, we plan to implement bootstrapping to obtain reliable standard errors when appropriate. If imbalances in participant characteristics are observed at baseline, we will consider propensity score weighting to improve efficiency [79]. We will explore missing data and apply standard practices to control for the differences between subjects in missing data or to impute scores if non-trivial levels of missing data are observed. Similar analytic methods will be applied to secondary outcomes, with appropriate adjustments based on the characteristics of the data and distributions. We will follow the CONSORT 2010 guidelines when reporting study results [80].

#### Mixed methods analysis

The interpretation of qualitative data from interviews will be considered in the context of quantitative data (intervention effectiveness results, intervention process/implementation statistics, HCW intervention acceptability ratings), as well as feedback, correspondence, and materials gathered from study partners throughout the study. This evaluation in context will illustrate the fit and feasibility of COMPASS-NP within states’ training models and HCW population. We will use these findings, combined with quantitative intervention effectiveness data to descriptively assess systems for replicating/replacing ergonomic tool vouchers, as well as between-system/state differences that may impede or encourage intervention accessibility, appropriateness, and adoption.

#### Qualitative data analysis

The narrative unit(s) to be analyzed will be bounded segments or episodes about an event within the interviews. We will follow four stages of analysis: (1) divide the text into episodes that comprise the plot/sequence of the story, (2) discard material irrelevant to the plot, (3) identify stanzas in each episode which comprise a single theme or embedded story, and (4) identify contrasts, binaries, and mediating terms (a blend of shared features) within and across each episode.

#### Treatment fidelity

Members of our research group will observe (not participate in) 20% of COMPASS-NP virtual meetings to score intervention fidelity (via a fidelity checklist) and take structured notes on discussion topics, participation, common questions/problems, and group dynamics. The study staff who observe a meeting will share the results of their observations with the group facilitator during a debrief and coaching conversation.

#### Data storage and security

A data safety and monitoring plan was developed in the original grant application to minimize risk and protect confidentiality. The grant application and the study protocol were reviewed and approved by the OHSU Human Subjects Institutional Review Board (IRB). This plan includes a system for implementing recording, appending, storing, and backing up data in accordance with IRB-approved procedures. The survey data for this project will be stored in the Oregon Clinical and Translational Research Institute’s (OCTRI) installation of REDCap, a highly secure and robust web-based research data collection and management system. Data will periodically be inspected for internal consistency, completeness, and quality. Rights to export data for processing and analyses are limited to the study team, and all exports are recorded and time-stamped by the REDCap system. Data exported for processing and analyses will be stored in the university’s secure cloud computing system with password-controlled access. In order to minimize the risk of confidentiality breach, each participant will be given a unique identifier number that will be used for entering data into electronic databases or labeling any other study materials collected from them. A separate file that links participant names to participant IDs will be stored separately from data exports and will be password-protected at the file level in addition to the folder access level.

### Timeline

The timeline from the original grant proposal for study activities aligned with each specific aim is provided in Table 1. Facilitators will be recruited and trained, and the virtual process and materials will be piloted. During years 2 through 4, we will recruit and train additional facilitators and implement the 3-wave cluster randomized waitlist control design with HCWs. In years 4 and 5, we will complete the final analyses and submit manuscripts for publication. Dissemination and outreach efforts will be ongoing throughout the grant. Process measures will include group facilitators participating in post-intervention phone interviews to provide feedback on implementation and their experiences during the study trial, web analytics from the COMPASS-NP webpage, and the online ergonomic self-assessment.

**Table 1:** COMPASS for navigating pain study timeline

**Timeline**		**YR 1**				**YR 2**				**YR 3**				**YR 4**				**YR 5**			
**A1**	Intervention translation	X	X	X	X			X													
	Facilitator recruit and train			X	X	X			X	X			X	X							
	Data processing/analysis					0	0														
**A2**	Implementation waves					X	X	X	X	X	X	X	X	X	X	X					
	Measures (0, 10,20 weeks)					X	X	X	X	X	X	X	X	X	X	X					
	Data processing/analysis								0	0			0	0		0	0	0	0	0	0
**A3**	Process measures					X	X	X	X	X	X	X	X	X	X	X	X				
	Process interviews								X				X				X	X			
	Data processing/analysis								0	0			0	0		0	0	0	0	0	0
**A4**	Dissemination/outreach						X			X				X		X	X	X	X	X	X

*X* implementations, *0* analyses and manuscripts

### Adverse events

Unanticipated events that place subjects or others at risk of harm or discomfort during the course of the study, and which are related to the study, will be reported immediately as an unanticipated problem. Harm to a subject need not occur for an event to be considered an unanticipated problem.

Any adverse event that occurs within the course of the study, either reported or observed, will be reported by the staff to Olson (PI) immediately via email or text/telephone. The research staff will complete an adverse events form to record the details about the event. This form will be delivered to Olson for further review within 2 days of the event. Olson will review all adverse events and determine whether they are unanticipated problems. Unanticipated problems will be communicated to the study sites, to the OHSU IRB via the OHSU eIRB system, and to the NIH Program Officer. Olson will notify the study sites/contacts of any unanticipated problems within 7 calendar days of learning of the event. The full adverse events plan is available from the OHSU IRB. If needed, accommodations will be made for any adverse events involving participants.

### Dissemination

COMPASS-NP trial knowledge and resources will be disseminated widely with guidance from the Principles of Good Dissemination from the National Institute for Health Research [81]. We will also employ the OHWC’s selected research dissemination model [82] that aims to optimize communication *Channels* for maximum impact by tailoring the information *Source* and *Message* for particular *Audiences*. Our first goal will be to disseminate the knowledge produced by the study to workers, labor organizations, government agencies, employers, and academia. These efforts are meant to enhance employer and academic knowledge of how to best address HCWs’ pain using a TWH-informed approach. Our second goal is to disseminate the COMPASS-NP toolkit. This assumes the intervention will be effective and appealing for organizations to adopt. While effectiveness is not established, our chances for success are strong given the original program’s appeal and research-to-practice success [28] and the evidence-based foundations of our translation plan. In this context, we expect COMPASS-NP dissemination to begin with negotiating and facilitating its adoption with our study partners and then marketing it to other home care organizations and stakeholders. Knowledge and intervention dissemination work is integrated throughout the study period. Depending on efficacy, we will disseminate COMPASS-NP in partnership with our University’s Technology Transfer Department.

## Discussion

COMPASS-NP will translate an established work-based safety and health program (COMPASS) to address the specific needs of HCWs experiencing chronic pain. We will add enhanced ergonomic protections and add adapted and new content for effective pain management, drawing from established CBT strategies for chronic pain self-management. These strategies are designed to prevent new injuries and re-injuries, reduce pain flare-ups, and reduce emotional distress and maladaptive pain responses (including opioid misuse). The tailored version of the intervention will leverage the peer-led and socially supportive structure of the original COMPASS program. The planned web-based group implementation approach will enhance accessibility for workers and will be evaluated with workers across three Pacific Northwest states. In total, COMPASS-NP will augment and strengthen original COMPASS materials to address an important secondary prevention need and opportunity for HCWs with chronic pain. The program’s primary target is to reduce pain interference with HCWs’ work and life domains and slow or halt the progression of pain and its many associated problems including work-related disability and opioid initiation and misuse. As a translational research project, study plans include integrated attention to knowledge and program dissemination to maximize long-term potential impacts.

### Trial status

Version 1.0 of the online ergonomic self-assessment tool has been developed using Adobe Articulate Software, based upon the NIOSH Handbook titled *Caring for Yourself while Caring for Others* [53]. This online tool guides users through a checklist of common HCW job tasks and then provides task-related ergonomic solutions in the form of low-tech tools and tips. Users will be able to select and receive low-tech ergonomic tools personalized to meet their work-related and high risk of injury activities (approximately $100 in value). Existing COMPASS scripted meetings are being adapted to address the needs specific to HCWs with chronic pain. For, example, exercises for workers with pain have been modified to provide less demanding exercises. New scripted meetings addressing self-management of pain are also in progress focusing on evidence-based CBT strategies. The randomized controlled trial is anticipated to begin in 2023 following the completion of pilot testing.

## Supplementary Information

**Additional file 1. **SPIRIT Statement Checklist.

## Data Availability

After the publication of findings linked to our specific aims as outlined in the research plan, requests for specific de-identified quantitative data from qualified individuals within the scientific community who have a published history of use of comparable datasets will be honored if the request is considered legal, credible, and with a scientific purpose and if a supporting Data Use Agreement is established by the involved universities. Requests for data can be initiated by contacting Ryan Olson: olsonry@ohsu.edu.

## Abbreviations

COMPASS-NP: COMmunity of Practice And Safety Support for Navigating Pain
HCW: Home care worker
NIOSH: National Institute for Occupational Safety and Health
SEIU: Service Employees International Union
OHCC: Oregon Home Care Commission
OHWC: Oregon Healthy Workforce Center
TWH: Total Worker Health®
